# P-907. Management of Corynebacterium striatum Deep-Seated Infections Across the Veterans Affairs Health Care System

**DOI:** 10.1093/ofid/ofaf695.1113

**Published:** 2026-01-11

**Authors:** Elena Stroman, Blake Remensnyder, Ashleigh Wallace-Lacey, Travis W Linneman, Ryan P Moenster

**Affiliations:** St. Louis Veterans Affairs Health Care System, St. Louis, Missouri; St. Louis Veterans Affairs Health Care System, St. Louis, Missouri; VA St. Louis HCS, St. Louis, Missouri; VA St. Louis HCS, St. Louis, Missouri; St. Louis College of Pharmacy at UHSP/VA St. Louis Health Care System, St. Louis, Missouri

## Abstract

**Background:**

*Corynebacterium striatum* is frequently isolated from deep tissue cultures, yet its clinical significance and optimal treatment remain uncertain.

**Methods:**

This was a retrospective cohort study of all patients in the Veterans Affairs Health Care System treated for *C.striatum* or non-speciated *Corynebacterium* isolated from ≥ 1 deep tissue culture between 1 January 2013 and 30 September 2022. Patients were assigned to 1 of 4 cohorts: 1) > 7 days treatment with a reliable agent (vancomycin, linezolid, tedizolid, lipoglycopeptide), 2) > 7 days treatment with an agent possessing variable activity (daptomycin, aminopenicillins [±β-lactamase inhibitor], doxycycline, minocycline, sulfamethoxazole-trimethoprim), 3) > 7 days treatment of an agent with no activity (all other antibiotics), and 4) definitive surgical source control with ≤ 7 days of antibiotics. The primary composite outcome was clinical failure, defined as microbiologic recurrence or antibiotic failure within 6 months, unplanned surgical procedure within 12 months, or 12-month all-cause mortality.

**Results:**

A total of 120 patients met inclusion criteria. Notable differences in baseline characteristics were ID consultation (94.9%, 82.1%, 77.3%, 40%), polymicrobial infection (56.4%, 76.9%, 54.5%, 45%), and *C.striatum* isolated (76.9%, 89.7%, 86.4%, 90%) in cohorts 1-4, respectively. Vancomycin was the most used antibiotic in cohort 1 at 82.1% followed by linezolid at 25.6%. Aminopenicillins (±β-lactamase inhibitor) were the most used antibiotic in cohort 2 at 41% followed by daptomycin at 35.9%.

Treatment failure occurred in 33.3% (13/39), 61.5% (24/39), 45.5% (10/22), and 65% (13/20) of patients in cohorts 1-4, respectively (p=0.018). When directly comparing cohort 1 against each individual cohort, treatment failure was significantly higher in cohorts 2 and 4 (p=0.019 and p=0.008, respectively). Multivariable analysis revealed receipt of a cohort 1 antibiotic was independently associated with a lower odds of treatment failure (OR 0.19, 95% 0.052-0.693). No other factors tested were associated with treatment failure.

**Conclusion:**

For deep-seated *C.striatum* and non-speciated *Corynebacterium* infections, treatment with reliable antibiotics was significantly associated with reduced risk of treatment failure.

**Disclosures:**

All Authors: No reported disclosures

